# Cross-Sectional Analysis of the Complaints and Emergency Department Attendances During COVID-19 Pandemic

**DOI:** 10.7759/cureus.12043

**Published:** 2020-12-12

**Authors:** Ali Ghaffar, Rajendar Garlapati

**Affiliations:** 1 Emergency Medicine, East Lancashire NHS Hospitals, Blackburn, GBR; 2 Accident and Emergency, East Lancashire NHS Hospitals, Blackburn, GBR

**Keywords:** accidents and emergency, a&e, covid-19

## Abstract

In December of 2019, an emergence of a new type of pneumonia resulted in a pandemic. To prevent the spread of coronavirus disease 2019 (COVID-19) caused by the severe acute respiratory syndrome coronavirus 2 (SARS-CoV-2), governments worldwide introduced a multitude of restrictions and preventative measures, including national lockdowns. Previous studies reported the clinical characteristics and epidemiology of the disease. The purpose of this article is to provide an analysis of changes in the number and nature of the accidents and emergency department attendances during the early phase of COVID-19 lockdown in the United Kingdom.

## Introduction

In December of 2019, a new atypical respiratory infection emerged in Wuhan, China, which attracted global attention. A novel virus was linked to multiple cases of pneumonia with marked potential for human-to-human infectivity. This disease was later termed coronavirus disease 2019 (COVID-19), and the pathogen was identified as severe acute respiratory syndrome coronavirus 2 (SARS-CoV-2) [1]. The disease rapidly spread worldwide. The World Health Organisation declared it a global pandemic on the 11^th^ of March 2020 [2]. Symptoms of the disease range from mild flu-like symptoms to acute respiratory distress syndrome (ARDS) and appear to vary depending on the age, ethnicity, and comorbidity status of the host. However, a significant proportion may remain asymptomatic [3, 4].

The continually rising death toll prompted various infection control measures to be introduced in different countries, including national lockdowns, social distancing enforcements, and the use of face coverings in public [5]. The disease remains an active concern at the time of writing, and emerging information has aimed to improve our understanding of the disease's pathophysiology and identify suitable cures. Published literature so far reviews clinical features and epidemiological characteristics of the disease. However, little information is available on the impact this pandemic has had on patient confidence in attending health services to treat new or existing health conditions.

## Materials and methods

Data was collected retrospectively using both triage and clinician clerk-in notes. All patients presenting to the emergency care departments for the months of April 2019 and April 2020 were included in the study. Data was compiled in the form of spreadsheets and analysed using data filters.

Our research focused on reviewing not only the number but also nature of presenting complaints during the month of April 2020 for Emergency and Urgent Care departments operating under the East Lancashire Hospitals NHS Trust. Geographically, the Trust serves a large area, including Blackburn, Darwen, and Burnley.

Furthermore, along with presenting original data, a comprehensive review was conducted of available literature [6-9]. Accordingly, this paper discusses how the available research collaborates and differentiates from the data outlined below. 

## Results

The overall number of attendances, attendances by gender, attendances by age, and variation in the nature of presenting complaint for the month of April 2020 was compared against similar data from April 2019 to evaluate the effects of the COVID-19 pandemic on service usage by the general public.

Total attendances

**Figure 1:**
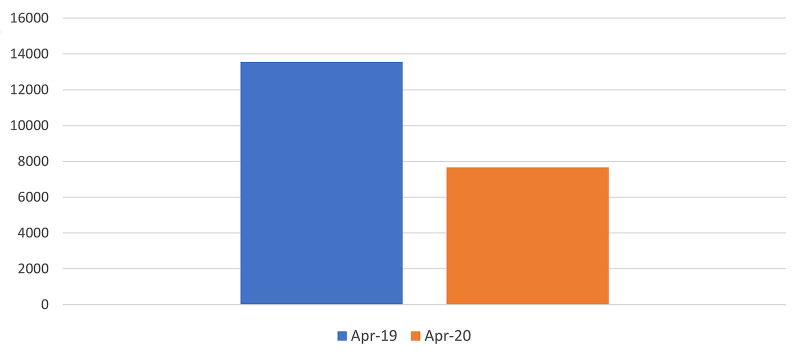
Total number of emergency department attendances in April 2019 compared to April 2020

Total attendances fell by 43.4% during April 2020 compared to the total number of attendances in April 2019 (Table 1, Figure 1)

**Table 1:** Total number of emergency department attendances in April 2019 compared to April 2020

Month	Patient numbers
April 2019	13,554
April 2020	7,673 (-43.4%)

Attendances by week of the month

**Figure 2:**
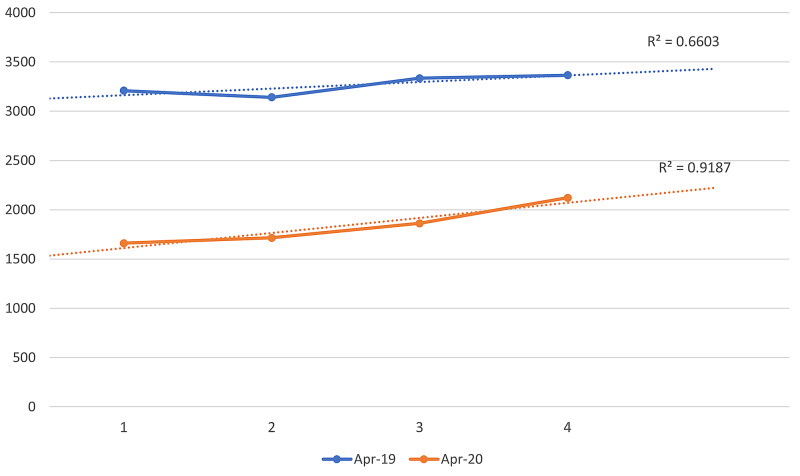
Total number of emergency department attendances by week of the month of April

Although overall attendances were down by nearly a half, we observed a gradual increase in attendances on a week-by-week basis during April 2020 (Table 2, Figure 2).

**Table 2:** Total number of emergency department attendances by week of the month of April

Week of April	April 2019	April 2020
1	3,208	1,661
2	3,141	1,716
3	3,337	1,863
4	3,366	2,121

Gender profile

The male to female attendance ratio remained grossly similar between April 2019 and April 2020 with a marginally higher male population attending the emergency department in April 2020 (Table 3).

**Table 3:** Total number of emergency department attendances by gender for April 2019 and April 2020

Gender	April 2019 (percentage of total)	April 2020 (percentage of total)
Male	6,789 (50.1%)	3,878 (50.6%)
Female	6,765 (49.9%)	3,793 (49.4%)

Age profile

**Figure 3:**
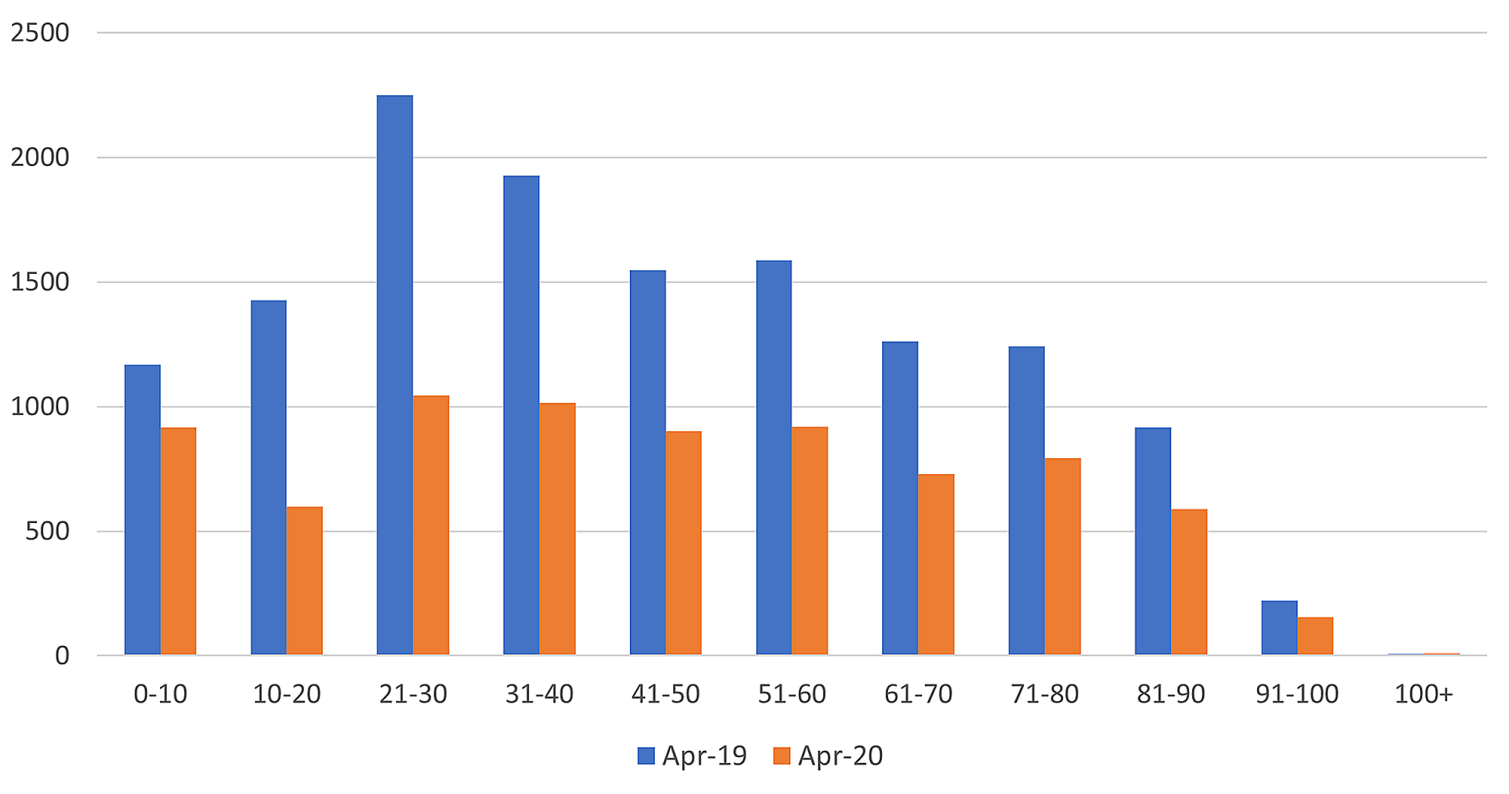
Net change of emergency department attendances by age groups for April 2019 and April 2020

**Figure 4:**
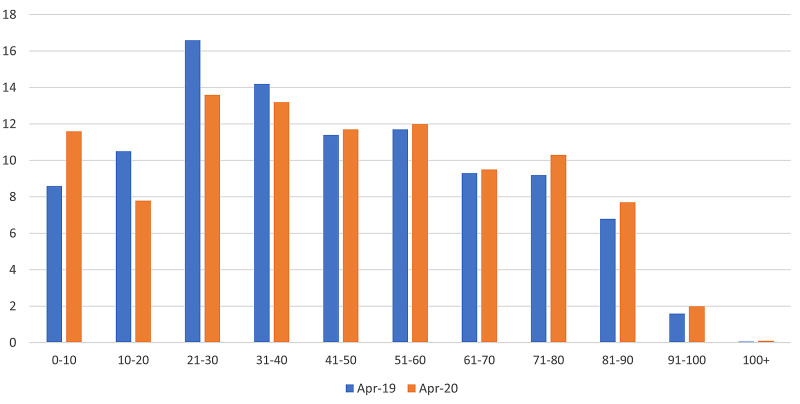
Percentage of total attendances to emergency department by age groups for April 2019 and April 2020

During April 2020, there was a net reduction in attendances across all age groups. However, a comparatively significant observation was made in the 21-30 age group. Data from April 2019 follows a normal distribution with the age group 21-30 being the most common age at presentation (Table 4, Figure 3, Figure 4). A sharp decline was seen in the 11-20 and 21-30 age groups attending the emergency departments during April 2020. In contrast, attendance by the 40+ age group was proportionally higher during April 2020.

**Table 4:** Total number of emergency department attendances by age groups for April 2019 and April 2020

Age group	April 2019 number of patients (percentage of total)	April 2020 number of patients (percentage of total)
0-10	1,169 (8.6%)	917 (11.6%)
11-20	1,426 (10.5%)	598 (7.8%)
21-30	2,250 (16.6%)	1,044 (13.6%)
31-40	1,927 (14.2%)	1,015 (13.2%)
41-50	1,547 (11.4%)	901 (11.7%)
51-60	1,587 (11.7%)	920 (12.0%)
61-70	1,261 (9.3%)	730 (9.5%)
71-80	1,241 (9.2%)	794 (10.3%)
81-90	917 (6.8%)	590 (7.7%)
91-100	221 (1.6%)	155 (2.0%)
100+	8 (0.06%)	9 (0.1%)

Discharge status

**Figure 5:**
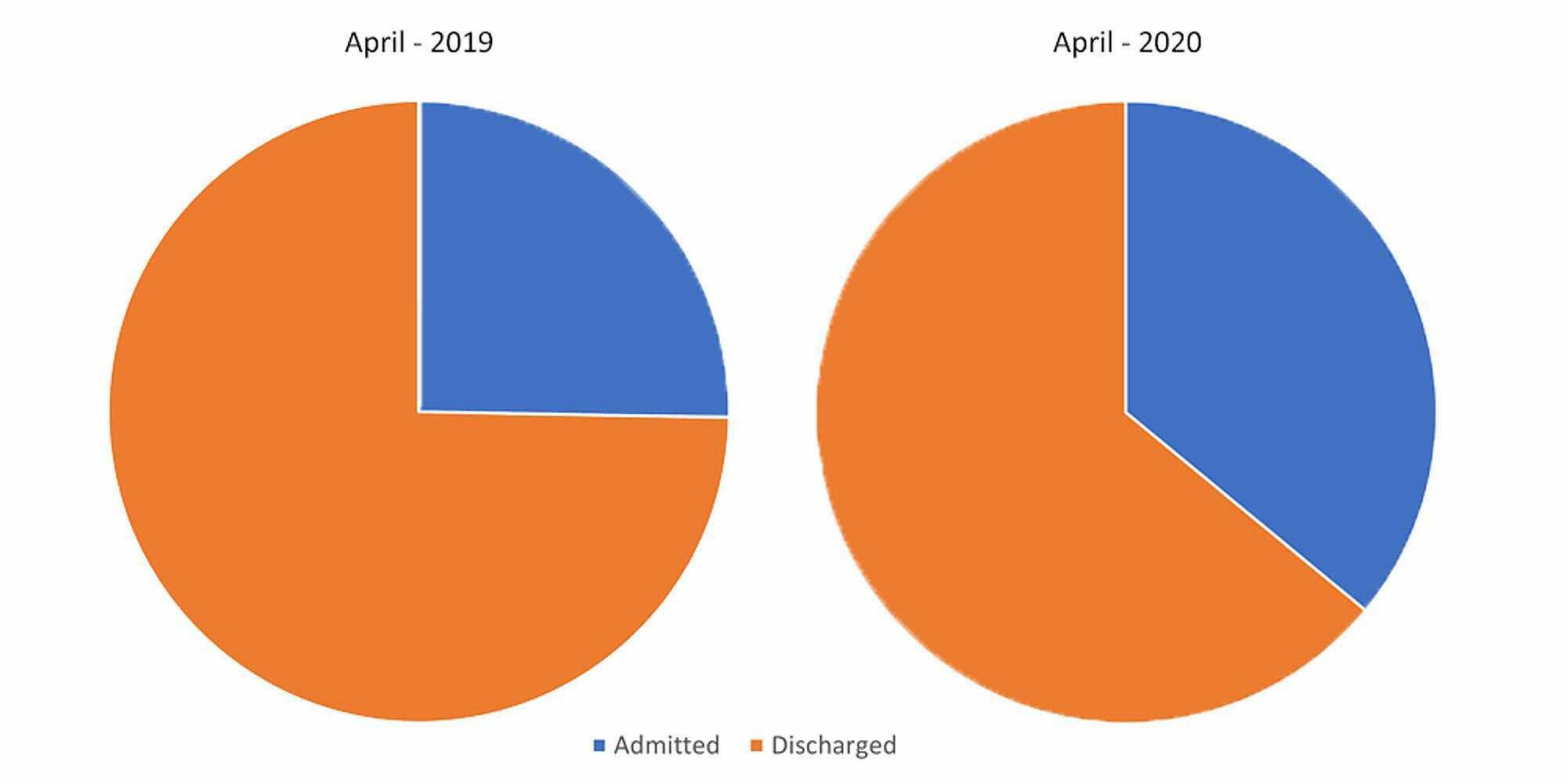
Discharge and admission status in April 2019 and April 2020

A relatively greater proportion of attending patients were admitted in April 2020 compared to April 2019 (Table 5, Figure 5).

**Table 5:** Total number of admissions and discharges during April 2019 and April 2020

	April 2019 (percentage of total)	April 2020 (percentage of total)
Admitted	3,428 (25.3%)	2,761 (36%)
Discharged	10,126 (74.7%)	4,912 (64%)

Presenting complaints

**Figure 6:**
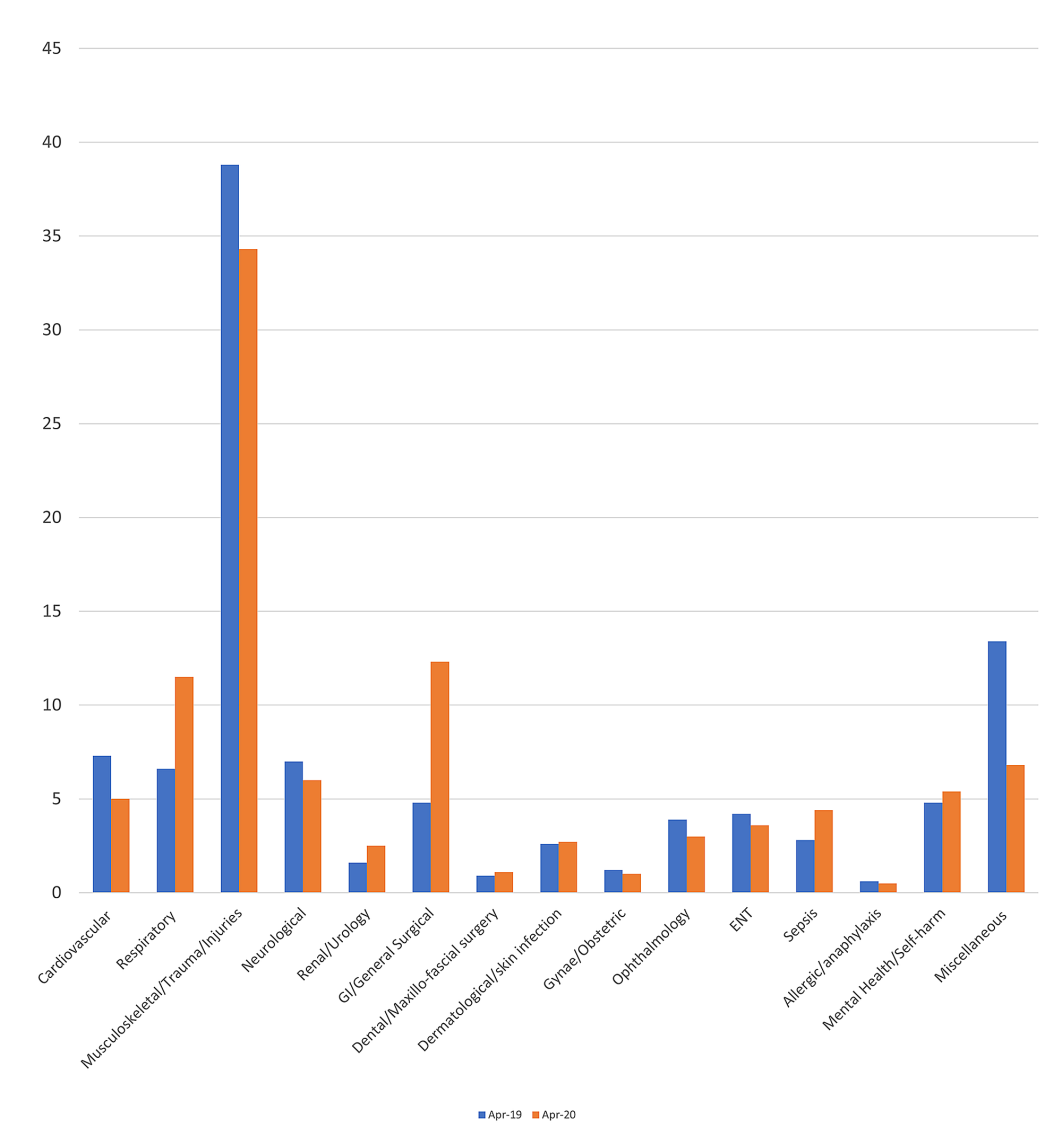
Percentage of total attendance by presenting complaint during April 2019 and April 2020 GI - gastrointestinal; ENT - ear, nose, throat

Musculoskeletal conditions, trauma and minor injuries were the most common presenting complaints during April 2019 and 2020. However, the number of attendances were lower in April 2020 (Table 6, Figure 6). When adjusted for the proportion of attendances, a decline was seen in patients with cardiovascular and neurological conditions.

**Table 6:** Total number of attendances by presenting complaint during April 2019 and April 2020 GI - gastrointestinal; ENT - ear, nose, throat

Presenting complaint	April 2019 number of patients (percentage of total)	April 2020 number of patients (percentage of total)
Cardiovascular	994 (7.3%)	380 (5.0%)
Respiratory	896 (6.6%)	884 (11.5%)
Musculoskeletal / trauma / injuries	5,261 (38.8%)	2,630 (34.3%)
Neurological	954 (7.0%)	460 (6.0%)
Renal / urology	211 (1.6%)	192 (2.5%)
GI / general surgery	645 (4.8%)	941 (12.3%)
Dental / maxillo-fascial surgery	118 (0.9%)	84 (1.1%)
Dermatological / skin infection	373 (2.6%)	210 (2.7%)
Gynaecology / obstetric	169 (1.2%)	76 (1.0%)
Ophthalmology	437 (3.9%)	230 (3.0%)
ENT	570 (4.2%)	280 (3.6%)
Sepsis	385 (2.8%)	336 (4.4%)
Allergic / anaphylaxis	75 (0.6%)	40 (0.5%)
Mental health / self-harm	655 (4.8%)	411 (5.4%)
Miscellaneous	1,811 (13.4%)	519 (6.8%)

There was a sharp rise in the number of attendances for respiratory conditions and sepsis. This may be attributed to the addition of COVID-19 infections-related burden but also due to the increased apprehension and concern among the general public, fearing that any respiratory symptoms may have been caused by COVID-19 infection.

Furthermore, a proportional increase was seen in patients presenting with dermatological, dental, gastrointestinal, and general surgical conditions. This could be attributed to reduced access to face-to-face GP and dental service appointments and the cancellation of elective surgical procedures throughout the country [10-12].

Finally, a proportionally higher number of mental health presentations potentially point towards the exacerbating effect of fear and concerns surrounding the COVID-19 infection and deaths as well as the reduction in opportunities to socialise and travel experienced during the lockdown as a potential contributing factor in affecting the mental health of the population.

## Discussion

The purpose of this study was to provide a cross-sectional analysis of emergency department attendances during the early phase of the COVID-19 pandemic. After outlining the data, the observations noted above will now be discussed with some possible explanations.

Firstly, there was a significant overall reduction in the number of attendances. It seems probable that much of this reduction in attendances to the emergency department was due to the fear of contracting SARS-CoV-2 among the general public. The fact that there was a gradual increase in attendance on a week-by-week basis can be explained by a number of factors such as an increasing number of COVID-19 infections or an increasing concern to seek medical help for any health condition. It could also be indicative of a possible gradual recovery in confidence in the general public.

Secondly, the decline in the 11-20 and 21-30 age groups attending the emergency care departments during April 2020 was observed. A possible explanation could be the reduced net human movement and fewer leisure opportunities during the lockdown resulting in fewer cases of musculoskeletal injuries amongst the younger population. The proportionally higher attendance by the 40+ age group during April 2020 could be explained by the likelihood of multiple co-morbidities in this age group requiring medical attention along with fewer face-to-face GP appointments available during the lockdown [10]. For most of the world’s population, particularly the younger generation, the restrictions on travel and ability to meet with other people outside of their household in order to prevent exposure to a dangerous disease was a new and potentially frightening experience. The highly infectious nature of the virus, along with poorly understood treatment options and near-constant media reporting of COVID-19 related deaths and admissions, may have heightened this fear.

Thirdly, the greater number of admissions to the hospital could be explained by a number of possible factors. Firstly, this could directly represent the increasing COVID-19 related admissions. Secondly, in the absence of an on-the-spot test for COVID-19, the unknown nature of the disease progression and overlapping symptoms of other pathologies could have resulted in uncertainty and apprehension in the admitting clinician fearing that any respiratory symptoms could, in fact, be due to COVID-19 and the patient’s condition may deteriorate further. This problem was compounded by our evolving understanding of the disease and resulting in frequent revisions in the local admission criteria for patients with respiratory compromise.

Higher relative admissions in April 2020 could also represent the cohort of patients who would have required admission regardless of the overall reduction in attendances. However, by that metric, a concerning 19.4% reduction in the admissions compared to April 2019 would represent the population that otherwise chose to nurse a potentially serious illness at home without seeking medical attention.

Finally, our data shows a significant reduction in cardiovascular and neurological related presentations. This reduction is a concern as the patients presenting with symptoms of cardiovascular or cerebrovascular ischemia require urgent investigations and potentially urgent reperfusion therapies. Delay or failure to receive treatment can result in high mortality and morbidity. It remains to be seen what proportion of stroke and myocardial infarction related deaths in the community can be attributed to the failure to seek help for fear of exposure to SARS-CoV-2. Increased public education to be vigilant for the pathognomonic symptoms of these serious conditions is important to prevent the avoidable delay in seeking help during further lockdowns.

The number of attendances for sepsis and respiratory conditions were proportionately higher. The data for the National Ambulance daily calls also shows an increased number of calls related to breathing problems and cardio/respiratory arrest during week 13 of the year 2020 [13]. This may be attributed to the increasing COVID-19 infection rate or an increased sense of awareness and a low threshold for seeking help for respiratory symptoms.

As noted above, other studies have similarly reported a profound reduction in the number of patients attending the emergency department during the lockdown and suggest patient reluctance as a possible reason in seeking help due to fear of contracting SARS-CoV-2 [6-9]. However, these studies largely focused on the reduction in acute coronary syndrome-related attendances. This study provides a much broader overview of the nature and magnitude of change in the attendance for multiple health condition categories that commonly present to the emergency department. Furthermore, this study compares observed variations in gender, age groups, and admission rates during the lockdown.

Limitations

Our study only focused on the data from a single NHS Trust. However, the findings from a large sample size representing a sizeable geographical area can likely be generalised elsewhere in the UK with appropriate adjustment for population density among other locally applicable variables.

Grouping patients under an umbrella category such as gastrointestinal/general surgery can limit the usability of the particular data set as it provides poor differentiation for the parent category of the presenting complaint making it difficult to evaluate changes with a high level of precision. However, the dataset serves to provide a snapshot comparative analysis of the change trends during the lockdown. Furthermore, with the evolving situation and availability of further data, this area will be a focus of further analysis.

## Conclusions

A marked reduction in emergency department attendances was observed during the early phase of the COVID-19 pandemic. Rather worryingly, much of this reduction was seen in conditions that may result in death and severe disability without timely treatment. At the time of writing, the COVID-19 situation is still evolving, and further lockdown measures are possible. Meanwhile, the data provides a useful proxy to the risk-benefit calculation that individual patients made in accessing healthcare services during the pandemics. It is hoped that these observations can be beneficial for the public health officials concerned with distributing resources and raising awareness to prevent unnecessary delays in presentations. Public health officials should consider educating the public about the risk of not seeking medical attention and address public fear of contracting the disease at attending the hospital. This can be achieved by increasing the visibility of the safety measures in place and enabling patients to practice these measures to reduce the disease's spread. 
